# Effects of mirror neuron system‐based training on rehabilitation of stroke patients

**DOI:** 10.1002/brb3.1729

**Published:** 2020-07-01

**Authors:** Huiwen Mao, Yan Li, Li Tang, Ye Chen, Jiawei Ni, Liang Liu, Chunlei Shan

**Affiliations:** ^1^ Department of Rehabilitation Medicine Tongren Hospital Shanghai Jiao Tong University School of Medicine Shanghai China; ^2^ School of Rehabilitation Science and Institute of Rehabilitation Medicine Shanghai University of Traditional Chinese Medicine Shanghai China

**Keywords:** cognitive function, mirror neuron system, stroke, training, upper extremity function

## Abstract

**Objective:**

To investigate the clinical effects of the mirror neuron system (MNS)‐based training on upper extremity motor function and cognitive function in stroke patients.

**Methods:**

Sixty stroke patients (time from stroke onset 3–9 months) with upper extremity paresis (Brunnstrom stage II–IV) and cognitive impairment (MoCA score ≥ 15) were enrolled in this study. Patients were randomly allocated into MNS treatment group (*N* = 30) and control group (*N* = 30). Both groups underwent regular training for upper extremity motor function and cognitive function, and the MNS group was trained with a therapeutic apparatus named mirror neuron system training (MNST) including different levels of action observation training (AOT). Training lasted 20 min/day, 5 days/week for 8 weeks. MoCA, reaction time, and Wisconsin Card Sorting Test (WCST) were assessed at baseline and 8 weeks after training. Furthermore, Fugl‐Meyer assessment (FMA) and Modified Barthel index (MBI) were adopted to evaluated upper extremity motor function and daily life ability.

**Results:**

After 8 consecutive weeks’ training, both groups showed significant improvements on the upper extremity motor function, cognitive function, and daily life ability score after training (*p* < .05). The MNS group showed significantly improved upper extremity motor function and cognitive function (*p* < .05) compared with control group.

**Conclusions:**

Combining MNS‐based and conventional training can improve upper extremity motor function and cognitive function in stroke patients.

## Graphical abstract

After 8 consecutive weeks’ training, both groups showed significant improvements on the upper extremity motor function, cognitive function, and daily life ability score after training (*p* < .05). The MNS group showed significantly improved upper extremity motor function and cognitive function (*p* < .05) compared with control group.Combining MNS‐based and conventional training can improve upper extremity motor function and cognitive function in stroke patients.

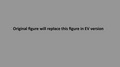

## 1 INTRODUCTION

Stroke is a common central nervous system disease characterized with loss of brain function, such as motor disorders, perception disorders, language disorders, and sensory disturbances. As the aging population increased, the incidence of stroke continuously rising. Studies showed that 75% of stroke patients suffer upper extremity dysfunction and 50% experience cognitive dysfunction (Huang & Yang, 2014; Serrano, Domingo, Rodriguez‐Garcia, Castro, & del Ser, 2007), which severely influence quality of life and places considerable burden on the patient's family and society. Therefore, the upper limb dysfunction combined with cognitive impairment are two important factors in daily living that should be focused on the rehabilitation field.

Current rehabilitation treatment approaches for poststroke cognitive dysfunction include transcranial magnetic stimulation, computer‐assisted cognitive function training system, virtual reality technology, and acupuncture treatment (Brooks & Rose, 2003; Cui, Ren, Du, Liu, & Zhang, 2014; Luber & Lisanby, 2014; Wang, Feng, et al., 2015). Treatments for improving upper extremity function include bilateral upper extremity training, gymnastic rod training, motor imagery (active visualization), robotics, functional electrical stimulation, electromyographic biofeedback, and proprioceptive neuromuscular facilitation(PNF) (Aisen, Krebs, Hogan, McDowell, & Volpe, 1997; Cauraugh & Kim, 2002; Iacoboni & Dapretto, 2006; Kilgore et al., 2003; Li & Tian, 2005; Liu & Feng, 2012; Ni et al., 2012; Xu & Wu, 2007). However, many patients suffer from both upper extremity and cognitive dysfunction following stroke, but the above mentioned rehabilitation techniques are insufficient to treat the two functional disorders simultaneously.

Mirror neuron system‐based training is one of the hot treatment technologies in recent years, which provides a motion‐observation‐execution matching mechanism and brings a new strategy for functional rehabilitation after stroke. In this study, we aim to further verify the effectiveness of mirror neuron‐based training (by a new apparatus named MNST) on both motor and cognitive function for 60 stroke patients over a relatively long time period (8 weeks).

## 2 MATERIALS AND METHODS

### 2.1 Patients

Our study has been approved by Ethics Committee of Shanghai Tongren Hospital, Shanghai Jiao Tong University School of Medicine. We also registered our clinical trial at www.chictr.ogr.cn after got ethics permission. The clinical trial registration number is ChiCTR 1800017588. Sixty patients were enrolled in this study and randomly divided into MNS group and Control group, with 30 patients in each group by a computer‐generated randomization list. All assessments in both groups were performed by a certain therapist, who did not treat these enrolled patients and also blinded to the treatment allocation. All patients had signed informed consent before enrollment.

### 2.2 Inclusion criteria

(a) First onset of stroke as confirmed with brain MRI, whose vital sign was stable and also with hemiplegia and cognitive impairment; (b) clinical course between 3–9 months; (c) age range from 40 to 80 years old; (d) signed informed consent and willing to attend our study; (e) right handed according to the Edinburgh Handedness Inventory; (f) >9 years of education (beyond junior middle school); (g) Brunnstrom stage II‐IV of the upper extremity; (h) MoCA scores of 15 or above.

### 2.3 Exclusion criteria

(a) Severe hypertension or cardiopulmonary disease; (b) Severe joint pain; (c) Patients with severe impairment of sight, hearing, and comprehension; (d) Deformity of upper extremity and hand; (e) People with diagnosed mental disorders.

### 2.4 Intervention method

**FIGURE 1:**
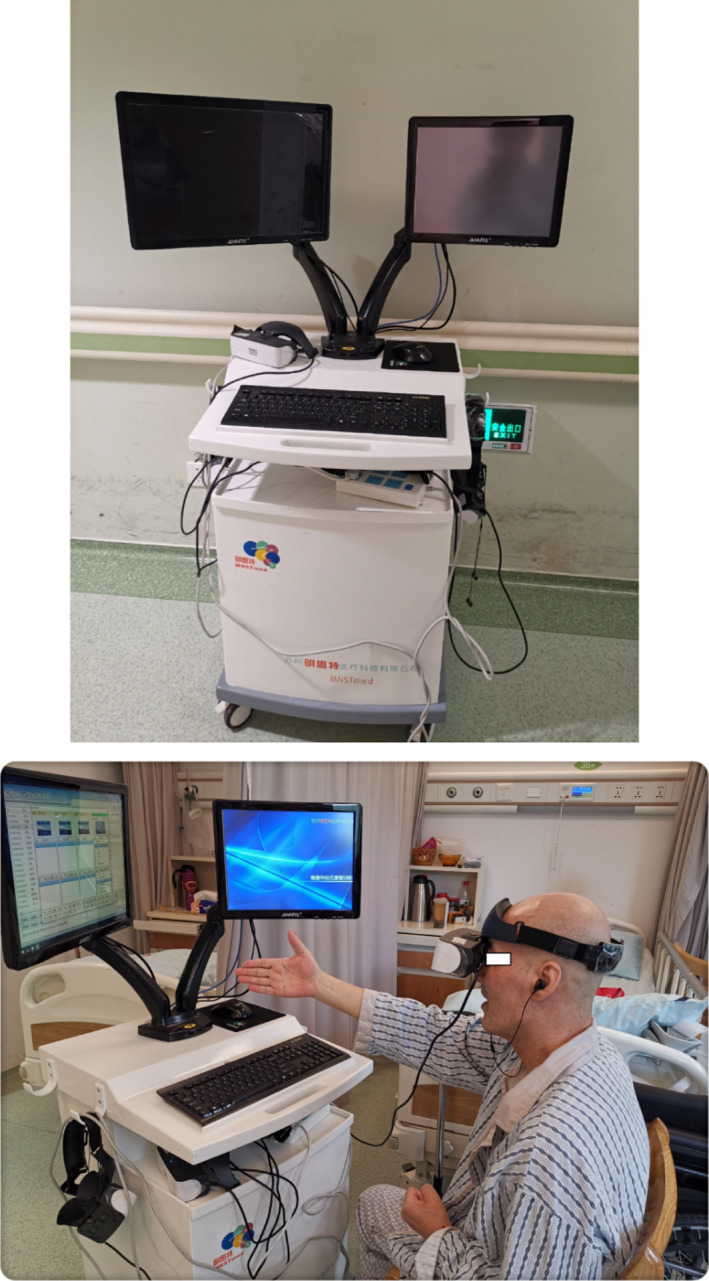
The Mirror Neuron System Training (MNST, V1.0, Suzhou MNST Medical Science and Technology Co., Ltd) machine and patient training status

A new apparatus named Mirror Neuron System Training (MNST, V1.0, Suzhou MNST Medical Science and Technology Co., LTD) based on mirror neuron theory was implemented to train the patients in this study (pictures shown in Figure 1).

Mirror neuron system training contains two primary parts: One involves virtual reality (VR) glasses, and the other is a system including hundreds of daily hand action videos, such as cracking a peanut, cutting a watermelon, and turning on an air conditioner. Using the VR glasses, the patients could see these hand action videos. This kind of action observation training (AOT) was reported to activate the mirror neuron system (overlap with motor, language, and cognition neural circuits) and therefore improve motor, language, and cognitive functions (Brooks & Rose, 2003; Cauraugh & Kim, 2002; Cui et al., 2014; Wang, Feng, et al., 2015).

### 2.5 Experimental procedures

The control group received routine upper limb rehabilitation training and Schulte Grid training as follows: (a) The placement of good limb position: mainly to inhibit the occurrence of spasm pattern. Adjust the position of affected side, uninjured side, and supine position every 2 hr. (b) Physical therapy (PT): mainly includes releasing shoulder joint, active, and passive scapula movement, inducing upper limb separation movement, improving abnormal muscle tension of upper limb, and improving control of upper limb movement. Sixty minutes once a day, 5 days per week, for 8 weeks. (c) Occupational therapy (OT): including activities involved hand manipulation and functional tasks for daily living. These activities were mainly targeted at upper limb function. They were pushing a ball, moving a plate, and simulated washing face, brushing teeth, eating, and dressing. 30 min once a day, 5 days per week, for 8 weeks. (d) Schulte Grid experiment: The Arabic numbers 1–25 randomly filled on 25 squares (each one is 1*1 cm). Patients were asked to pick out the number in order of 1–25 by their fingers and read aloud at the same time. 30 min per time once a day, 5 days per week, for a total of 8 weeks.

The MNS group received the same upper limb rehabilitation training as well as the mirror neuron‐based cognitive training by the MNST V1.0. The participants were asked to wear the glasses to watch the 40 hand motion videos chosen by the therapist in advance and imitated the actions in video at the same time. Each video was automatically set to play 3 times, and each training was 20 min. A total of 40 video were observed, once a day, 5 times a week for 8 weeks.

Routine upper limb rehabilitation training, Schulte Grid training, and Mirror Neuron System training were performed at home. Routine upper limb rehabilitation training was performed by a certain physical therapist. And another two were performed by a cognitive therapist, both of them were blinded to the treatment allocation.

### 2.6 Outcome measurement

Patients were assessed at baseline and after 8‐week treatment. The evaluations included: MoCA, simple reaction time, Wisconsin Card Sorting Test (WCST) were adopted to assess the cognitive function. It included Categories Completed (CC), Total Errors (TE), Perseverative Errors (PE), Nonperseverative Error (NRPE). Furthermore, Fugl‐Meyer assessment (FMA), Modified Barthel index (MBI) were used to assess the upper extremity motor function and daily life ability.

### 2.7 Statistical analysis

All data were analyzed with SPSS 22.0 software (IBM, Inc.). The paired‐sample *t* test was used if the data were normally distributed, and the Wilcoxon signed‐rank test was used if the data were not normally distributed. Sample size and power calculations were performed prior to undertaking the study to determine the number of participant's needed in each group with the PASS (Power Analysis and Sample Size) software. The calculations were based on detecting a mean difference of 20 clinically important difference on FMA assuming a standard deviation of 20, a two‐tailed test, an alpha level of 0.05, and a desired power of 90%. The estimated desired sample size was 26 individuals per group.

MoCA, simple response, FMA, and improved Barthel index results before and after treatment between the two groups were compared by an independent sample *t* test and paired *t* test within each group. Statistical significance was determined when *p* < .05.

## 3 RESULTS

### 3.1 General information

**FIGURE 2:**
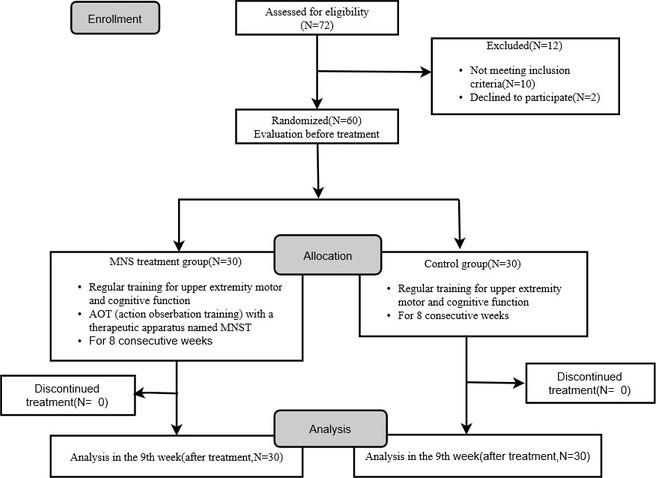
Study Flowchart

From September 2016 to July 2017, 72 stroke patients transferred to our Rehabilitation Department. Both groups were well tolerated, and no adverse event occurred during the study. Figure 2 illustrates the participants flow. Twelve patients were excluded before baseline assessment. During 8‐week study period, no patient dropped out. There are 30 participants in control group and 30 participants in MNS group.

In control group, there were 23 infarcts and 7 hemorrhages. While there were 24 infarcts and 6 hemorrhages in MNS group. Baseline demographic characteristics between groups including sex proportion, the average of age, education level, clinical course, and NIHSS score had no significant difference (Table 1). The MoCA, simple response time, FMA, MBI, and WCST (CC, TE, PE, NRPE) were not statistically different between the two groups (Tables 2 and 3).

**Table 1:** Clinical baseline information of participants(gender, age, clinical course, NIHSS score, stroke type, education level) (Mean ± *SD*)

Group	*n*	Gender		Age (year)	Clinical course (month)	NIHSS score	Stroke type		Education level
		M	F				Infarct	Hemorrhage	
MNST group	30	16	14	54 ± 7	6 ± 2	5 ± 3	23	7	12 ± 3
Control group	30	15	15	57 ± 6	6 ± 2	5 ± 3	24	6	12 ± 3
*p* value				.145	1.000	.965	.754		.535
*T* value				−1.476	0.000	−0.045	0.098		0.624

Note

The two groups were compared using unpaired *t* tests.

**Table 2:** Clinical baseline information of participants before and after treatment (The MoCA, simple response, FMA, and MBI)

Group	MoCA		Simple Response Time		FMA		MBI	
	Before	After	Before	After	Before	After	Before	After
MNS group (*n* = 30)	22 ± 6	28 ± 2	0.9 ± 0.1	0.5 ± 0.1	20 ± 11	46 ± 8	33 ± 10	52 ± 7
Control group (*n* = 30)	22 ± 5	24 ± 4	0.9 ± 0.1	0.8 ± 0.1	19 ± 11	33 ± 10	34 ± 11	46 ± 10
*p* value	.911	.000	1	.000	.72	.000	.755	.017
*T* value	−0.112	4.156	0	−10.454	0.36	5.709	−0.31	2.452
95% confidence interval	(−3.1,2.8)		(−0.1,0.7)		(−4.5,6.5)		(−6.2,4.5)	

Note

The two groups were compared using unpaired *t* tests.

**Table 3:** Comparison of the Wisconsin Card Sorting Test (WCST) between the two groups before and after treatment

Group	Categories Completed (CC)		Total Errors (TE)		Perseverative Errors (PE)		Nonperseverative Error (NRPE)	
	Before	After	Before	After	Before	After	Before	After
MNS group (*n* = 30)	2.9 ± 0.5	5.8 ± 0.8	80 ± 1	61 ± 1	44 ± 1	30 ± 1	36 ± 2	31 ± 1
Control group (*n* = 30)	3.0 ± 0.9	4.6 ± 0.8	80 ± 1	76 ± 1	44 ± 1	38 ± 1	36 ± 2	38 ± 1
*p* value	.458	.000	.523	.000	.166	.000	.683	.000
*T* value	−0.748	5.691	−0.642	−58.325	1.403	−30.789	1.440	22.031
95% confidence interval	2.900 ± 0.548	5.767 ± 0.817	80.033 ± 1.189	61.200 ± 1.064	44.367 ± 1.402	29.733 ± 1.258	35.673 ± 1.953	31.472 ± 1.279

Note

The two groups were compared using unpaired *t* tests.

### 3.2 Effects compared between the two groups

The participants in both groups had improvement at the end of 8‐week training. Moreover, MNS group showed greater improvements in upper limb function and quality of life such as FMA (*p* = .000) and MBI (*p* = .017). (Table 2).

Participants in MNS group also had obvious improvement in cognitive function including MoCA (*p* = .000), simple reaction time (*p* = .000), WCST (CC, TE, PE, NRPE) (*p* = .000) (Tables 2 and 3).

## 4 DISCUSSION

The incidence of stroke has increased in recent years, with subsequent dysfunctions cognitive, swallowing, and motor ability commonly observed. With such high prevalence, loss of activity of daily living (ADL), and heavy family and societal burden (Buccino, Solodkin, & Small, 2006; Zhang & Ma, 2013), cognitive and upper extremity dysfunction are primary goals and challenges in stroke rehabilitation.

Several new treatment techniques such as action observation training (AOT) are based on the mirror neuron system (MNS) theory. MNS involves an action observation‐ execution matching mechanism that encompasses visual observation, motor imagination, imitation, and learning. This process stimulates neural plasticity by activating the brain MNS system following stroke.

Discovery of the mirror neuron is one of the most important advances in the field of neuropsychology. Mirror neurons will fire both when executing movement (e.g., hand movement) and observing the same movement (Luber & Lisanby, 2014). Mirror neurons are therefore considered an important neural substrate for understanding action, imitation, language learning, and empathy. The mirror neuron system (MNS) primarily consists of the inferior frontal gyrus (BA44), premotor cortex (BA6), and inferior parietal lobule (BA39, 40) of the brain (Luber & Lisanby, 2014). Mirror neurons system activation has been found to contribute to improved motor functions in stroke patients (Sale & Franceschini, 2012; Small, Buccino, & Solodkin, 2012), as well as language and spatial attention functions of stroke patients with aphasia and hemineglect (Chen et al., 2015; Wang, Zhang, et al., 2015). Therefore, MNS‐based training may potentially be a valuable new therapeutic strategy for functional recovery after stroke (Zhang & Ma, 2013). However, most studies regarding MNS‐based training for stroke have assessed small sample sizes observed the effects after short periods of training (e.g., 3–4 weeks). Furthermore, few studies reported concurrent motor and cognitive function recovery following MNS‐based training for stroke patients.

In the present study, we trained poststroke patients in utilizing their upper extremities and test cognitive disorders through AOT. The goal was to confirm the efficacy of this new training program on motor and cognitive impairment for patients following stroke.

The results demonstrated that the MNS group exhibited improved MoCA, simple reaction time, FMA, modified Barthel index(MBI), and Wisconsin Card Sorting Test (WCST) outcomes over the control group. This indicated that activation of the MNS by AOT positively impacted motor and cognitive recovery for these patients. This fits with the observations that repeated action observation training activates the MNS located in the lower part of precentral gyrus, posterior inferior frontal gyrus, inferior parietal lobule, and superior temporal gyrus, where advanced brain function such as motor control and cognition occur. Many studies have shown that MNS in these areas is also associated with understanding movement and touch perception, and repeated stimulation promotes remodeling of the cerebral cortex, thus promoting the recovery of impaired brain function (Iacoboni & Dapretto, 2006; Liu & Feng, 2012; Rizzolatti & Craighero, 2004).

We also found improvements in Wisconsin Card Sorting Test (WCST) results, which indicate that AOT treatment improved patients’ concentration and ability for mental multitasking. The regions involved in these activities are located in the prefrontal cortex and temporal gyrus, which coincide with the areas of MNS distribution. Based on the results of the present study, we propose that treatment stimulating the MNS simultaneously activated these areas, improving aspects of patients’ cognition. Concentration is an important part of cognitive function and plays a role in functional recovery of the upper extremities. We clinically noticed that patients with attention disorders could not understand and cooperate with the therapist. As a result, the effect of rehabilitation training was often very poor. Therefore, it is essential to improve concentration ability and attention span to aid in therapeutic rehabilitation and promote effective motor relearning.

To the best of our knowledge, this is the first report on the effectiveness of mirror neuron‐based training (by a new apparatus) on both motor and cognition function in a cohort of stroke patients over a relatively long period of time (8 weeks). In addition, this therapeutic model is in accordance with the current popular "central‐peripheral‐central" closed‐loop rehabilitation model, which conforms to the latest hand function and cognitive rehabilitation trends.

Due to the restriction of sample size, site, and equipment, our study was unable to provide mechanistic evidence concerning the relevant roles of different periods of onset in patients. Another limitation is the lack of follow‐up because of not allowed so long‐term evaluation, which may be prone to biases.

Future studies will further be established to address and resolve these questions.

## 5 CONCLUSION

Combining MNS‐based and conventional training can improve upper extremity motor function and cognitive function in stroke patients.

## CONFLICT OF INTEREST

None declared.

## AUTHOR CONTRIBUTIONS

HM, YL, and CS designed the study; HM, LT, LL, and JN performed the clinical management and data collection; HM and YC performed the data analysis; HM wrote the manuscript draft; and all authors reviewed the manuscript.

## Data Availability

All original data will be available when contact the corresponding authors by email.
